# The Recombinant Lactobacillus Strains with the Surface-Displayed Expression of Amuc_1100 Ameliorate Obesity in High-Fat Diet-Fed Adult Mice

**DOI:** 10.3390/bioengineering11060574

**Published:** 2024-06-06

**Authors:** Xueni Zhang, Lei Jiang, Cankun Xie, Yidi Mo, Zihao Zhang, Shengxia Xu, Xiaoping Guo, Ke Xing, Yina Wang, Zhijian Su

**Affiliations:** 1 Guangdong Provincial Key Laboratory of Bioengineering Medicine, Department of Cell Biology, Jinan University, Guangzhou 510632, China; 2 School of Life Sciences, Guangzhou University, Guangzhou 510655, China; 3 National Engineering Research Center of Genetic Medicine, Jinan University, Guangzhou 510632, China

**Keywords:** Amuc_1100, cell-surface display, *Lactobacillus rhamnosus*, *Lactobacillus plantarum*, high-fat diet, intestinal health

## Abstract

Excessive dietary fat intake is closely associated with an increased risk of obesity, type 2 diabetes, cardiovascular disease, gastrointestinal diseases, and certain types of cancer. The administration of multi-strain probiotics has shown a significantly beneficial effect on the mitigation of obesity induced by high-fat diets (HFDs). In this study, Amuc_1100, an outer membrane protein of *Akkermansia muciniphila*, was fused with green fluorescent protein and LPXTG motif anchor protein and displayed on the surface of *Lactobacillus rhamnosus* (pLR-GAA) and *Lactobacillus plantarum* (pLP-GAA), respectively. The localization of the fusion protein on the bacterial cell surface was confirmed via fluorescence microscopy and Western blotting. Both recombinant strains demonstrated the capacity to ameliorate hyperglycemia and decrease body weight gain in a dose-dependent manner. Moreover, daily oral supplementation of pLR-GAA or pLP-GAA suppressed the HFD-induced intestinal permeability by regulating the mRNA expressions of tight junction proteins and inflammatory cytokines, thereby reducing gut microbiota-derived lipopolysaccharide concentration in serum and mitigating damage to the gut, liver, and adipose tissue. Compared with *Lactobacillus rhamnosus* treatment, high-dose pLR-GAA restored the expression level of anti-inflammatory factor interleukin-10 in the intestine. In conclusion, our approach enables the maintenance of intestinal health through the use of recombinant probiotics with surface-displayed functional protein, providing a potential therapeutic strategy for HFD-induced obesity and associated metabolic comorbidities.

## 1. Introduction

Obesity is a chronic, relapsing, and progressive disease that has emerged as an important global public health concern. Clinical evidence unequivocally demonstrates that obesity constitutes a primary risk factor for numerous non-communicable diseases, including coronary heart disease, hypertension, stroke, various cancers, type 2 diabetes, gallbladder diseases, lipid abnormalities, osteoarthritis, gout, and pulmonary diseases [1,2,3]. Consequently, individuals affected by obesity typically experience a reduction in life expectancy ranging from approximately 3.3 to 18.7 years compared to those within a normal weight range [4]. According to the World Obesity Federation report, the global population of overweight or obese individuals is anticipated to escalate from 38% to 50% between 2020 and 2035. This rise is anticipated to elevate the prevalence among adult males from 14% to 23% and among adult females from 18% to 27% [5]. Notably, overweight and obesity are more prevalent in males than females [6]. These conditions markedly increase healthcare expenditures, imposing a substantial financial burden on nations and governments, thereby impeding social and economic progress [6,7]. It is estimated that the economic ramifications of overweight and obesity will lead to potential income losses exceeding USD 4.32 trillion, approaching 2.9% of the global gross domestic product (GDP) by 2035 [5].

The characteristics of gut microbiota are intricately linked to the onset and progression of obesity and metabolic diseases [8]. Through a comparison between germ-free and normal mice, scientists identified, for the first time, that the microbiota possesses the capacity to promote the absorption of monosaccharides, leading to the induction of hepatic lipogenesis [9]. Subsequent studies further established that an imbalance in the composition of gut microbiota contributes to obesity by influencing energy metabolism and inflammation [10,11]. It has been confirmed that the abundance of *Akkermansia muciniphila*, a member of the Verrucomicrobia phylum, is inversely correlated with overweight, obesity, and type 2 diabetes in both rodents and humans [12,13,14]. Intriguingly, both live and pasteurized *A. muciniphila* have demonstrated the ability to reduce fat mass development, insulin resistance, and dyslipidemia in mice [15,16]. Further investigation indicated that Amuc_1100, a membrane protein of *A. muciniphila*, may mediate the beneficial effects of *A. muciniphila* by improving the gastrointestinal barrier and restoring gut microbiota abundance via the Toll-like receptor 2 pathway [16,17]. 

As the substrate for microbial metabolism, diet emerges as the primary factor influencing the community structures and species composition of the gut microbiota. Our previous investigation revealed a significant reduction in body weight, particularly among male mice, following a dietary switch, accompanied by the rapid reshaping of the gut microbiota within a short timeframe [18]. Sequencing data indicated a notable increase in the abundance of *A. muciniphila* in mice experiencing weight loss compared to the control group, while the abundance of *Lactobacillus spp.* decreased significantly. Lactic acid bacteria (LAB) are recognized for their involvement in lipid metabolism through the regulation of gut microbiota and enhancement of intestinal permeability [19]. Several LAB strains, including *Lactobacillus reuteri*, *Lactobacillus casei*, *Lactobacillus acidophilus*, *Lactococcus lactis*, *Lactobacillus gasseri*, *Lactobacillus paracasei*, and *Lactobacillus plantarum*, have demonstrated efficacy in improving obesity in clinical trials [20,21,22,23,24,25,26]. Furthermore, probiotic mixtures as dietary supplements exhibit distinct metabolic benefits compared to single probiotic preparations [24,27]. 

Most Lactobacillus strains serve as promising recombinant hosts for delivering functional proteins to mucosal tissues, given their ability to survive harsh conditions and colonize intestinal tissues [28]. Based on the aforementioned results, the primary objective of the current study was to investigate the feasibility of utilizing a recombinant Lactobacillus strain expressing Amuc_1100 for the treatment of obesity. Amuc_1100 was constitutively expressed, fused with an LPXTG motif that covalently anchored to the cell wall and green fluorescence protein (GFP), displaying on the surface of *Lactobacillus plantarum* (*L. plantarum*) and *Lactobacillus rhamnosus* (*L. rhamnosus*), respectively. The efficacy of these recombinant strains against obesity was assessed through oral administration in an adult mouse model subjected to a high-fat diet.

## 2. Materials and Methods

### 2.1. Reagents and Strains

Information regarding plasmids, bacterial strains, culture media, antibodies, quantitative polymerase chain reaction (qPCR) primers, and primers for constructing the expression cassette can be found in Supplementary Table S1. 

### 2.2. Experimental Animals

Male C57BL/6J mice (at postnatal day 60, 20–22 g/each) were purchased from the Experimental Animal Centre of the Guangdong Province (Guangzhou, China). The animals were housed in the animal facilities of the Jinan School of Medicine under controlled light conditions (12 h light, 12 h dark) with free access to food and water. The animal experiment protocol was approved by the Institutional Animal Care and Use Committee of Jinan University and was in accordance with the NIH Guide for the Care and Use of Laboratory Animals.

### 2.3. The Construction of Recombinant Expression Vectors

**Figure 1:**
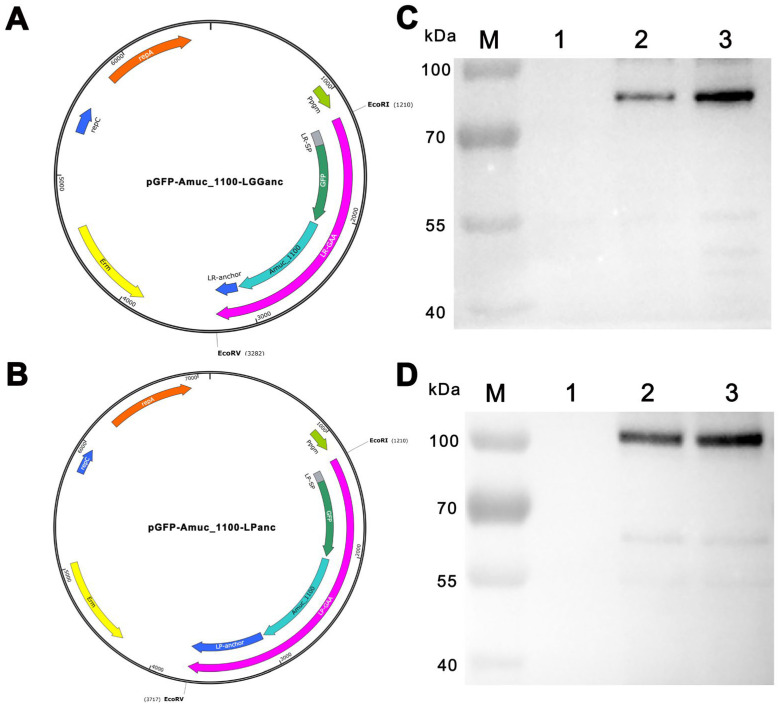
Schematic overview of recombinant vectors and Western blot analysis of fusion proteins. (**A**) The recombinant vector diagram depicting the cell-surface display of the Amuc_1100 fusion protein on *Lactobacillus rhamnosus* ATCC 53103 (*L. rhamnosus*). The fusion protein consists of *L. rhamnosus*-derived signal peptide, GFP, *Akkermansia muciniphila*-derived Amuc_1100, and *L. rhamnosus*-derived LPXTG motif anchor protein. The DNA fragment encoding Amuc_1100 fusion protein was cloned into constitutive expression vector pTRK892 using E*co*R I and E*co*R V sites to form the recombinant expression vector pGFP-Amuc_1100-LGGanc. (**B**) The recombinant vector diagram depicting the cell-surface display of the Amuc_1100 fusion protein on *Lactobacillus plantarum* ATCC 8014 (*L. plantarum*). The fusion protein consists of *L. plantarum*-derived signal peptide, GFP, Amuc_1100, and *L. plantarum*-derived LPXTG motif anchor protein. (**C**) Western blot analysis of fusion protein LR-GAA. Lane M represents a protein molecular marker; Line 1 represents *L. rhamnosus* harboring an empty vector; Lines 2–3 represent two pLR-GAA transformants. (**D**) Western blot analysis of fusion protein LP-GAA. Lane M represents a protein molecular marker; Line 1 represents *L. plantarum* harboring an empty vector; Lines 2–3 represent two pLP-GAA transformants. Ppgm: the phosphoglycerate mutase promoter; rep C: replicon C; rep A: replicon A; erm: erythromycin; kDa: Kilodaltons.

The schematic illustration of the domain organization for Amuc_1100 fusion protein was presented in Figure 1A,B. The selection of the secretion signal peptide and the cell wall-anchoring domain were referred from the previous studies for *L. plantarum* [29] and *L. rhamnosus GG* [30], respectively. The synthetic genes encoding the GFP-Amuc_1100-Anchor fusion proteins were synthesized according to *Lactobacillus* spp. preferred codon usage and then cloned into pTRK896 to create recombinant expression vectors, pGFP-Amuc_1100-LPanc and pGFP-Amuc_1100-LGGanc. The accuracy of the inserted synthetic gene was confirmed via DNA sequencing. The recombinant vectors were transformed into *L. plantarum* (ATCC 8014) and *L. rhamnosus GG* (ATCC 53103) through electro-transformation and are henceforth referred to as pLP-GAA and pLR-GAA, respectively. The transformed cells were selected on MRS agarose plates containing 150 μg/mL erythromycin and incubated at 37 °C for 2–3 d. The control plasmid, pTRK892, and the corresponding transformants, referred to as pLP-Con and pLR-Con, were also obtained through the methods described above. 

### 2.4. The Detection of Fusion Protein with Fluorescence Microscopy

The single clone of the transformants was cultured in 5 mL of MRS broth, containing 150 μg/mL of erythromycin, and incubated at 37 °C without shaking for 3 d. Cells from 100 μL of culture were harvested, resuspended in PBS, and captured using Leica DM6000 (Leica Microsystems, Wetzlar, Germany) fluorescence microscopy.

### 2.5. Quantitative Real-Time Polymerase Chain Reaction (qRT-PCR)

For mRNA qRT-PCR, the total RNAs from the cells and tissues were extracted and used as templates for cDNA synthesis. The reverse transcriptase reactions contained 400 ng of total RNA, 4 μL of 5× buffer, 2 μL of 10× nucleic acid mix, 2 μL of reverse transcriptase mixture, and nuclease-free water. The cDNA was diluted 1:3, and 2 μL of the diluted template was used per 20 μL of the qRT-PCR assay. All PCRs were performed using a Bio-Rad CFX Connect Real-Time system (Bio-Rad Laboratories, CA, USA), and the data were collected using the Bio-Rad CFX Manager software (version 2.0). The relative expression levels of the targeted mRNAs were normalized against the expression of *rpl19*. The fold changes of the expression between the treatments and controls were calculated using the 2^−ΔΔCt^ method. The efficiency of qRT-PCR performance for target genes was between 100–105%. The primers are listed in Supplementary Table S2. All data were derived from three different independent experiments.

### 2.6. Western Blotting

The cells were lysed in a lysis buffer. An equal number of total proteins was loaded on a 15% sodium dodecyl-sulfate polyacrylamide gel electrophoresis (SDS-PAGE). The proteins in the gels were transferred to a polyvinylidene difluoride (PVDF) membrane and blocked with a blocking solution containing 5% non-fat dry milk. Then, the membranes were incubated with primary antibodies for 12 h at 4 °C. After washing with Tris-buffered saline solution containing 0.5% Tween-20 (TBS-T) five times, the membranes were incubated with horseradish peroxidase (HRP)-conjugated secondary antibodies at room temperature for 1.5 h. The membranes were then washed with TBS-T five times and subjected to enhanced chemiluminescence (ECL) detection. The protein expression in tissues was normalized to the expression of β-actin. 

### 2.7. Hematoxylin and Eosin (H&E) Staining

The samples, including gut, liver, and fatty tissue, were fixed 24 h in 10% formalin solution and then dehydrated in gradually increasing concentrations of ethanol. Subsequently, the slices were deparaffinized using xylene and ethanol and rehydrated using distilled water. After staining for 3 min in Mayer Hematoxylin solution, the slides were rinsed with distilled water and then stained in an Alcoholic-Eosin solution for 1 min. 

### 2.8. Oral Glucose Tolerance Test (OGTT)

OGTTs were conducted in 8 h-fasted animals by measuring blood glucose levels at t = 0, 15, 30, 60, 90, and 120 min from a tail capillary blood sample using a hand-held glucometer (The Accu-Chek Performa blood glucose meter, Roche Diabetes Care GmbH, Shanghai, China) following an oral administration of glucose (40% dextrose) at a dose of 2 g/kg of body weight. 

### 2.9. The Measure of LPS Concentration

The quantitative determination of LPS in serum was measured using the LPS ELISA kit according to the instructions. Briefly, the standards, controls, and serum samples (100 µL, in duplicate) were dispensed into numbered wells, and incubated for 2 h at 37 °C. A total of 100 µL of Biotin-antibody was added to each well and incubated for 1 h at 37 °C. Each well was aspirated and washed 3 times using wash buffer. Subsequently, 100 µL of HRP-avidin was added to each well and incubated for 1 h at 37 °C. After adding 90 µL of substrate, the optical density of each well was measured using a microplate reader at 450 nm. The sensitivity of this assay system was 0.039 ng/mL. The intra-assay and inter-assay variations were less than 8 and 10%, respectively. The results from all samples (*n* = 10) were averaged for the statistical analysis.

### 2.10. Animals and Treatment

For the *L. rhamnosus GG* experiment, 60 C57BL/6J male mice were randomly divided into six groups (*n* = 10 per group): control, model, pLR-Con treatment, pLR-GAA treatment (recombinant *L. rhamnosus GG* three dose levels). The recombinant *L. rhamnosus* cultures were freshly harvested, washed, and resuspended with saline every day. Each animal in the control group and model group was given a daily oral administration of 150 μL of saline. The three pLR-GAA dose levels were high (1 × 10^9^ CFU/d), medium (1 × 10^8^ CFU/d), and low (1 × 10^7^ CFU/d) and indicated by pLR-GAA-H, pLR-GAA-M, and pLR-GAA-L, respectively. The pLR-Con group was given pLR-Con daily at a 1 × 10^9^ CFU dose level. The anti-obesity experiment was performed for 8 weeks. The mice belonging to the model, pLR-Con treatment, and pLR-GAA treatment groups were fed with a high-fat diet (60% energy from fat). The mice in the control group were fed with standard chow (10% energy from fat). Weight gain, dietary intake, and blood glucose levels were measured once a week. After finishing the oral glucose tolerance test, the animals were killed using carbon dioxide suffocation. The blood and tissues were collected and stored at −80 °C for the subsequent determination. For the evaluation of *L. plantarum*, the bacterial preparation and the experimental procedures were identical to those described above. 

### 2.11. Statistical Analysis

The mice were randomly assigned to the treatment or control groups. Basic pairwise comparisons were performed with independent sample *t*-tests and a one-way ANOVA was performed for group comparisons. Within the time-point pairwise assessments of the groups, differences were rendered in terms of 95% confidence intervals to convey effect sizes and their patterns over time. All analyses were performed using Prism (version 9.0; GraphPad, La Jolla, CA, USA) software and the data are expressed as the means and standard errors of the means (means ± S.E.M.). The differences are regarded as significant at *p* < 0.05, *p* < 0.01, and *p* < 0.001.

## 3. Results

### 3.1. Construction, Expression, and Detection of Recombinant Fusion Proteins

**Figure 2:**
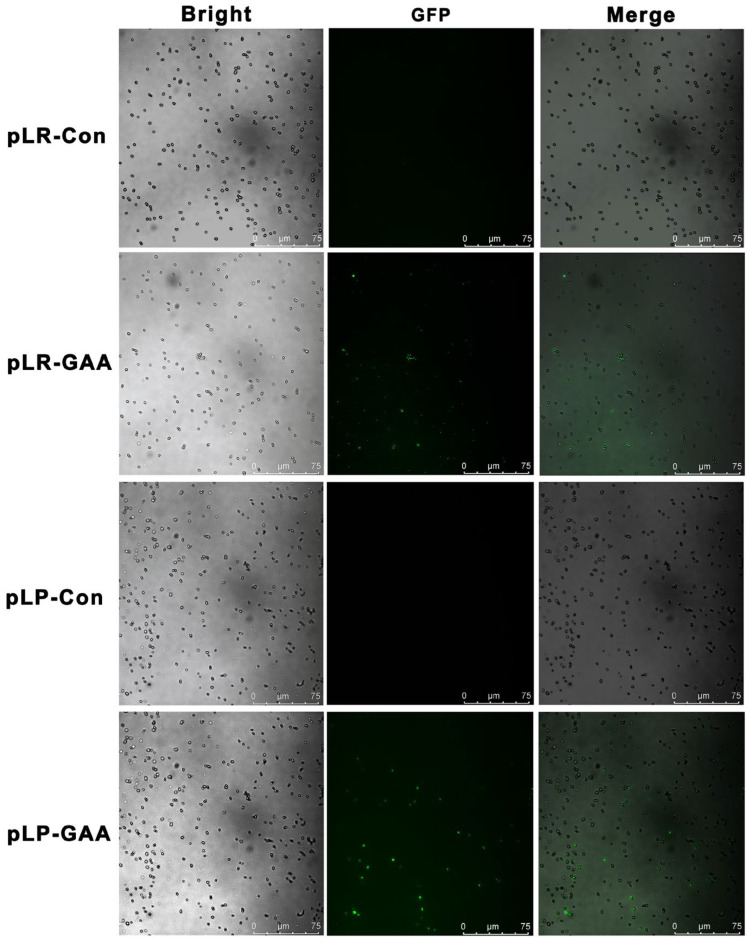
The morphological observation of recombinant Lactobacillus strains. The recombinant transformants pLR-GAA and pLP-GAA as well as the control strains pLR-Con and pLP-Con were cultured for 3 d, harvested, and observed using fluorescence microscopy.

The fusion protein displayed on the cell surface was composed of a secretory signal peptide, GFP, Amuc_1100, and an LPXTG motif anchor protein. The structural domains of this fusion protein, specifically the segments between GFP and Amuc_1100 as well as between Amuc_1100 and the LPXTG motif, were linked via a glycine-rich sequence (Supplementary Tables S3 and S4). DNA fragments encoding these fusion proteins were cloned into the constitutive expression vector pTRK896 using E*co*R I and E*co*R V restriction endonuclease sites, as illustrated in Figure 1. Subsequently, the vectors were transformed into recombinant *L. plantarum* and *L. rhamnosus*, yielding the recombinant pLP-GAA and pLR-GAA, respectively. After 3-d incubation, the recombinant cells were collected and lysed via homogenization. The debris containing the cell wall was subjected to treatment at 100 °C for 5 min and analyzed with Western blotting. The results confirmed that both fusion proteins, indicating the correct molecular weight as expected, reacted positively with the anti-GFP antibody (Figure 1C,D). Both fusion proteins, with the expected molecular weights, were capable of reacting positively with the anti-GFP monoclonal antibody (Figure 1). Fluorescence microscopy was employed to localize the fusion protein expression, revealing that after a 3-d incubation, the cell surfaces exhibited green fluorescence, in contrast to the non-fluorescent control (Figure 2). These findings demonstrate the successful cell surface display of the Amuc_1100 fusion proteins in a full-length pattern on *L. plantarum* and *L. rhamnosus*.

### 3.2. Recombinant Lactobacillus Strains Showed Beneficial Effects on the HFD-Induced Hyperglycemia

**Figure 3:**
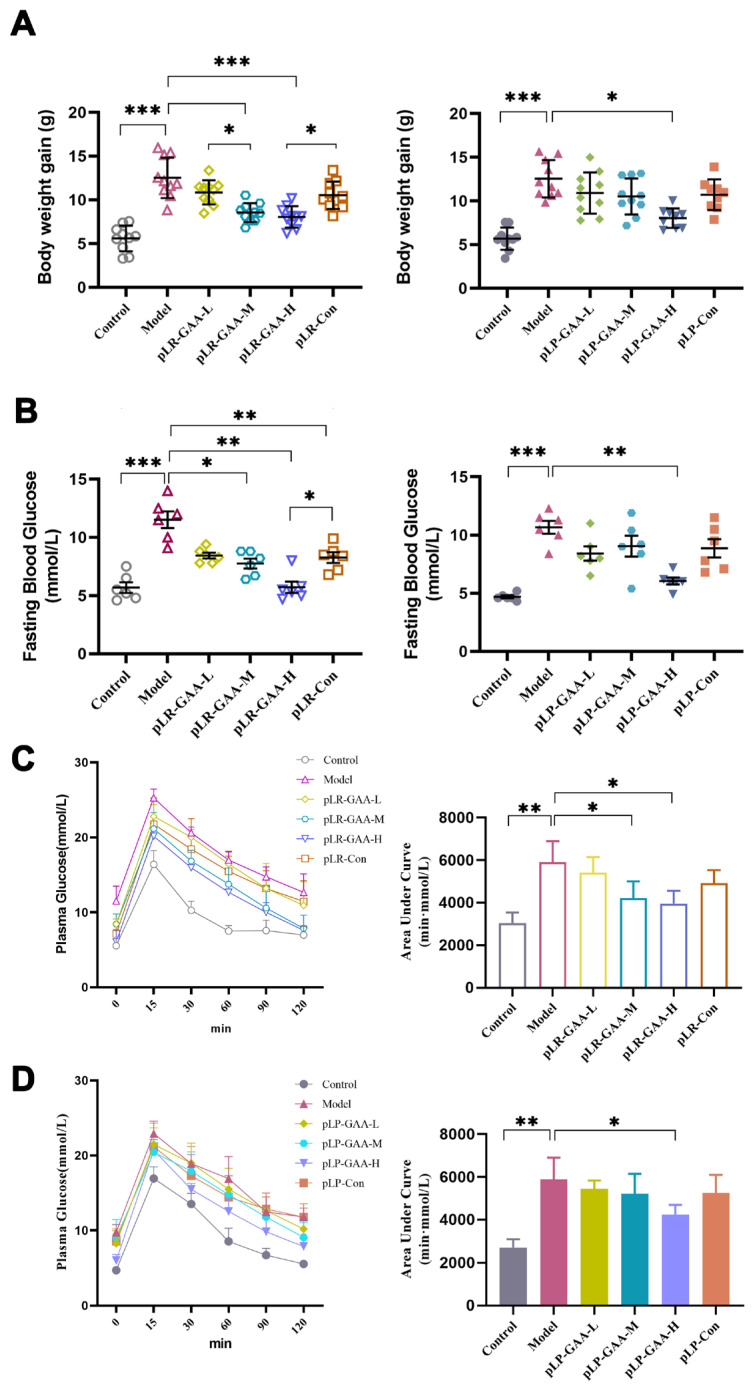
The functional assay of recombinant Lactobacillus strains in HFD-treated mice model. The mice were randomly divided into six groups. Each animal in the control group and model group was given a daily oral administration of 150 μL of saline. In treatment groups, each mouse was given recombinant bacterium at 1 × 10^9^ CFU/d, 1 × 10^8^ CFU/d, and 1 × 10^7^ CFU/d, respectively. The pLR-Con and pLP-Con groups were given pLR-Con and pLP-Con daily at a 1 × 10^9^ CFU dose level, respectively. (**A**) The analysis of body weight gain after administration of 8 weeks (*n* = 10; * *p* < 0.05, *** *p* < 0.001). (**B**) The analysis of fasting blood glucose concentration (*n* = 6; * *p* < 0.05, ** *p* < 0.01, *** *p* < 0.001). (**C**,**D**) Blood glucose levels analysis during an oral glucose tolerance test (OGTT) performed in 8 h-fasted mice (*n* = 6; * *p* < 0.05, ** *p* < 0.01).

Next, we conducted an evaluation of the preventive effects of recombinant Lactobacillus strains on HFD-induced obesity. After daily administration for 8 weeks, the average weight gain in the model group was 12.08 ± 2.45 g, which was 2.64 times higher than that in the control group. In the group treated with recombinant *L. rhamnosus*, the administration of pLR-GAA to mice fed a high-fat diet resulted in a significant reduction in body weight gain in a dose-dependent manner. Specifically, the average body weight gains of pLR-GAA-H, pLR-GAA-M, and pLR-GAA-L were reduced by approximately 40.64%, 36.92%, and 9.35%, respectively, relative to the model group. The positive control, treated with pLR-Con (1 × 10^9^ CFU/d), exhibited a reduction of approximately 16.56% compared to the model group. Comparable improvements were obtained in mice treated with recombinant *L. plantarum*, with the average body weight gains for the pLP-GAA-H, pLP-GAA-M, and pLP-GAA-L treatments decreasing by approximately 23.18%, 18.96%, and 14.16%, respectively, in comparison to the model group (Figure 3A). 

High-fat diets induce hyperglycemia and insulin resistance in rodents and humans. Therefore, we assessed the potential preventive effects of recombinant Lactobacillus strains on metabolic disorders. Eight weeks following the initiation of a high-fat diet, the model group of mice exhibited an average fasting blood glucose (FBG) level of 11.52 ± 0.88 mmol/L, representing a 2.08-fold increase compared to the control group, which had an FBG of 5.54 ± 0.51 mmol/L. The administration of pLR-GAA significantly mitigated hyperglycemia in a dose-dependent manner, with the high-dose pLR-GAA treatment achieving FBG levels comparable to those of the control group. The positive control, pLR-Con (1 × 10^9^ CFU/d), was able to partially restore glucose homeostasis, achieving a 26.4% reduction in FBG compared to the model group, although this level remained significantly elevated in comparison to the control group (Figure 3B). In the cohort treated with recombinant *L. plantarum*, the average FBG levels were 4.87 ± 0.23 mmol/L and 10.70 ± 0.49 mmol/L for the normal and the model groups, respectively. No significant differences in FBG were found between the model group and the groups treated with pLP-Con, pLP-GAA-L, and pLP-GAA-M. Only the high-dose pLP-GAA treatment was effective in reducing hyperglycemia, with treated mice showing an FBG of 6.13 ± 0.28 mmol/L (Figure 3B). Further, the oral glucose tolerance test confirmed that the middle- and high-dose pLR-GAA, as well as the high-dose pLP-GAA treatments, protected the animals from hyperglycemia induced by a high-fat diet (Figure 3C,D). Notably, there were no statistically significant differences in HFD consumption between the model and experimental groups, suggesting that the beneficial effects of recombinant Lactobacillus strains on body weight gain and blood glucose were not attributable to reduced food intake.

### 3.3. The Analysis of Histomorphology and the Expression of Inflammatory Factors in Gut 

**Figure 4:**
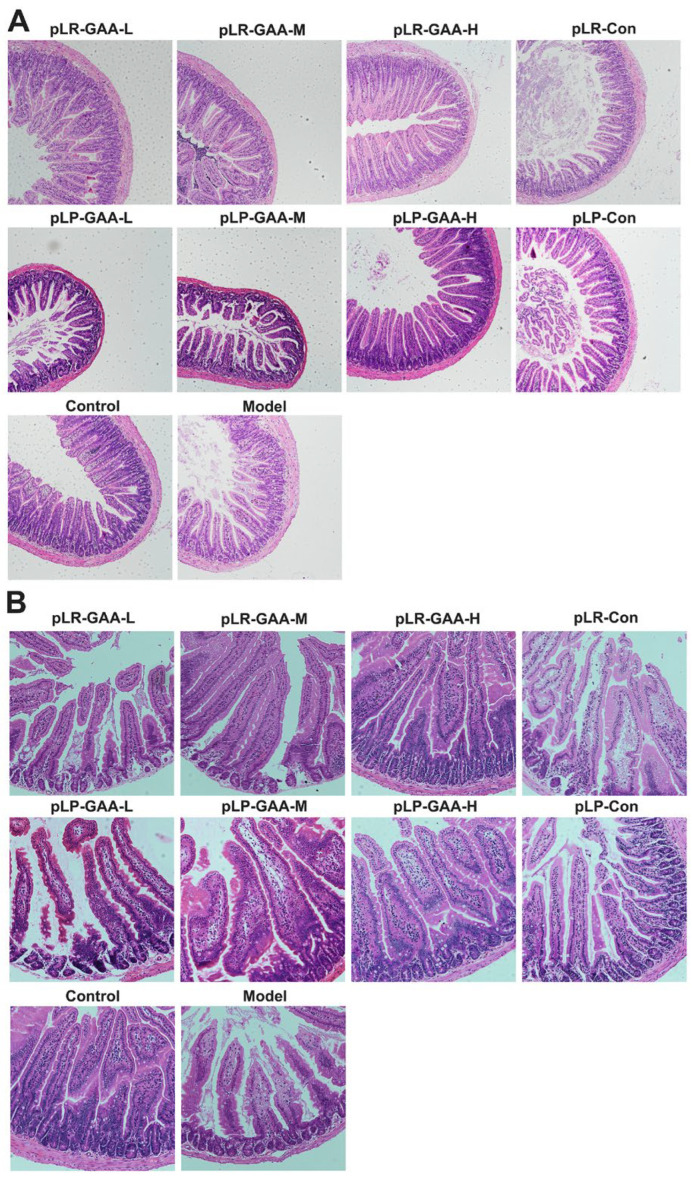
The histomorphological analysis of mouse intestinal tissue. (**A**) The ileum (magnification 100×); (**B**) the Jejunum (magnification 200×).

Long-term HFD impairs the integrity of the gut barrier; therefore, we investigated the intestinal histomorphology and the expression of inflammatory and tight junction proteins. A histological analysis of the ileum and jejunum, conducted via Hematoxylin and Eosin (H&E) staining, revealed that villi in the control group were elongated, numerous, and densely arranged with intact structural integrity. Conversely, in the high-fat diet-treated group, villi appeared sparsely distributed and were significantly reduced in both density and number. Treatment with low-dose recombinant Lactobacillus strains exhibited no notable improvement in comparison to the model group. However, the middle-dose treatment groups demonstrated a denser arrangement of villi and a partial restoration of normal morphological characteristics, with the high-dose treatment yielding the most pronounced protective effects in terms of villi number and arrangement. The control treatments, pLR-Con and pLP-Con, showed enhancements in villi number and arrangement relative to the model group, yet they did not achieve the structural integrity observed in the normal control group (Figure 4A,B).

**Figure 5:**
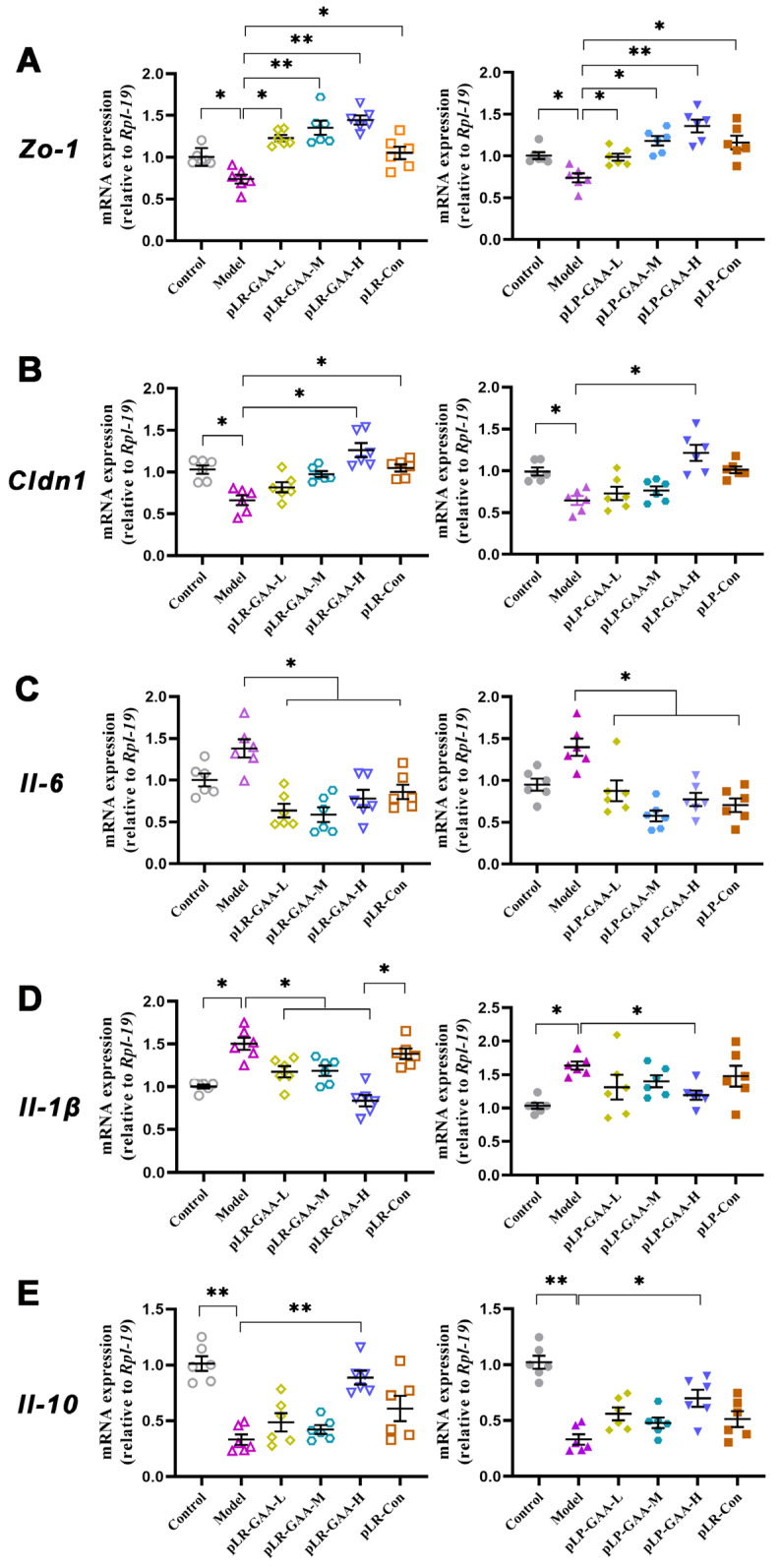
The mRNA expression of tight-junction genes and inflammatory genes in intestinal tissue. (**A**) *Zo-1*; (**B**) *Cldn-1*; (**C**) *Il-6*; (**D**) *Il-1β*; and (**E**) *Il-10*. All quantitative data were obtained from three independent experiments and presented as means ± S.E.M.; * *p* < 0.05; ** *p* < 0.01.

The qPCR results indicated a significant downregulation in the expressions of zona occludens-1 (*Zo-1*) and claudin-1 (*Cldn1*) in the model group. The supplementation of recombinant Lactobacillus strains resulted in a dose-dependent upregulation of these genes in the intestinal tissue of the animals. The expression levels of *Zo-1* were elevated to 2.0- and 1.8-fold higher than those of the model group in the pLR-GAA-H and pLP-GAA-H treatment groups, respectively. Within the pLP-GAA treatment cohort, only the high-dose pLP-GAA and pLP-Con were effective in restoring the expression of *Clnd1* (Figure 5A,B). 

**Figure 6:**
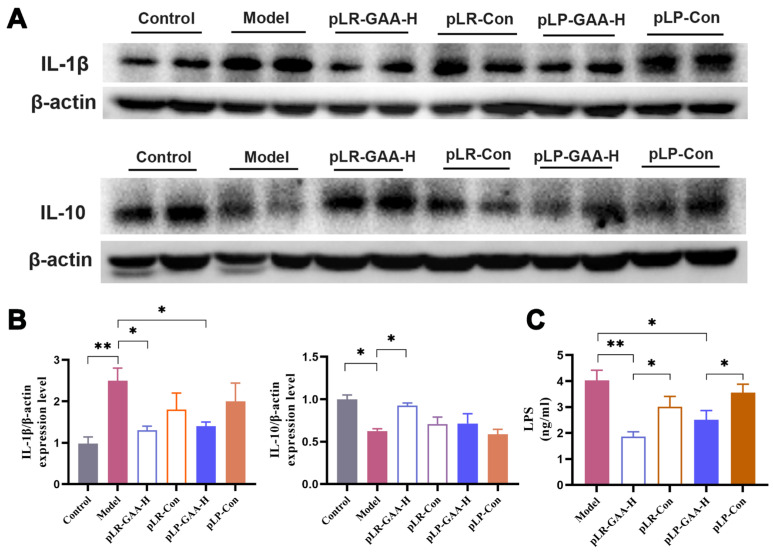
The assessment of inflammatory characteristics in intestine and serum. (**A**) Representative Western blotting for IL-1β and IL-10 expression. (**B**) Semiquantitative analysis of IL-1β and IL-10 levels, which were normalized to β-actin levels. (**C**) The concentration of LPS analyzed by ELISA. The Western blot band intensities were measured with ImageJ software. The data are presented as means ± S.E.M.; * *p* < 0.05; ** *p* < 0.01.

Furthermore, an HFD has been demonstrated to increase intestinal permeability by elevating cytokines that disrupt the gut barrier, particularly Interleukins. In the model group, the expressions of *Il-1β* and *Il-6* were significantly elevated, whereas *Il-10*, an anti-inflammatory cytokine, was diminished in comparison to the normal group (Figure 5C–E). The treatments with high-dose pLR-GAA and pLP-GAA effectively mitigated the HFD-induced increases in *Il-1β* and *Il-6*. Notably, while treatments with other high doses exhibited a tendency to enhance IL-10 expression, only the high-dose pLR-GAA treatment succeeded in restoring the expression of IL-10 in mRNA and protein levels (Figure 6A,B).

### 3.4. Effects of Recombinant Lactobacillus Strains on the Histomorphology of Liver and Adipose Tissues

**Figure 7:**
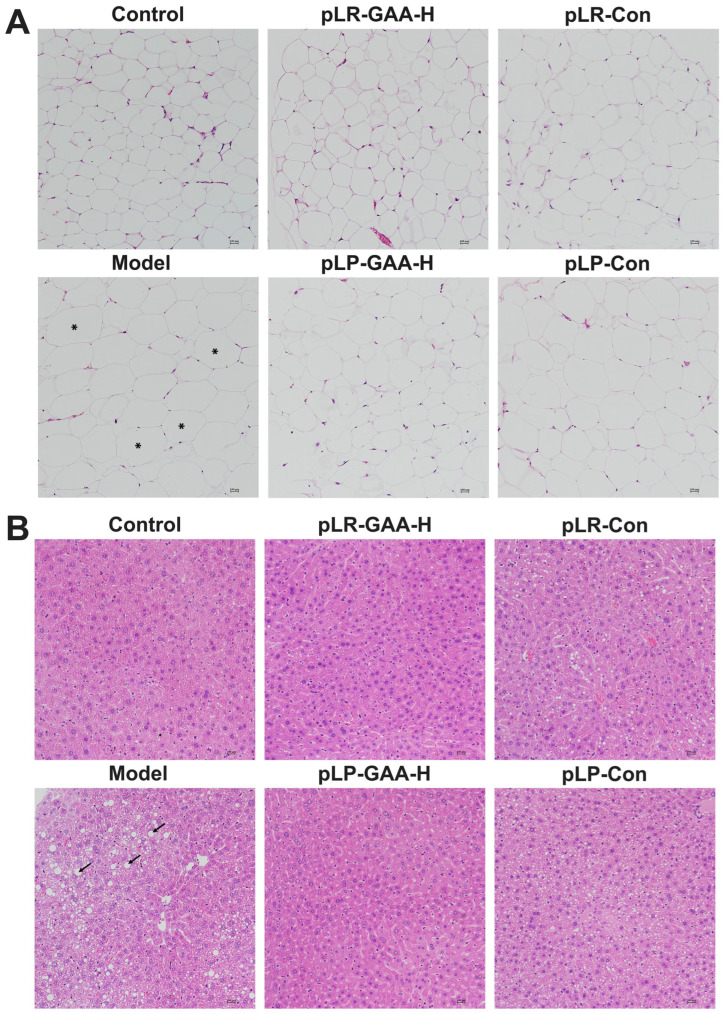
The morphological comparison of the liver and epididymal fat tissue with H&E staining. The high-dose recombinant Lactobacillus strains alleviated damage to the liver and epididymal fat tissue in HFD-fed mice. (**A**) The epididymal adipose tissue (magnification 200×, scale bar 100 μm). The star markers represent the enlarged adipocytes. (**B**) The liver tissue (magnification 200×, scale bar 100 μm); the long arrows represent the lipid vacuoles.

Alterations in gut permeability have been associated with an increase in the concentration of lipopolysaccharides (LPSs) in the serum, which can induce low-grade inflammation and subsequently contribute to obesity and related metabolic disorders [31]. In the model group, the average concentration of LPSs was 4.03 ± 0.24 ng/mL. The supplementation of high-dose pLR-GAA and pLP-GAA resulted in a significant decrease in serum LPS concentrations to 1.76 and 2.51 ng/mL, respectively (Figure 6C). The histological analysis of epididymal adipose tissue showed that mice fed a high-fat diet had larger adipocytes compared to those in the control group. Remarkably, the administration of high-dose pLR-GAA and pLP-GAA led to a notable reduction in the size of adipocytes (Figure 7A). Furthermore, the staining of liver tissue from the model group revealed a disorganized arrangement of hepatocytes with diffuse fatty degeneration, characterized by the presence of variably sized, nearly spherical vacuoles. Conversely, treatment with Lactobacillus strains at high doses significantly diminished the presence of liver tissue vacuoles and lipid droplets, resulting in a more compact arrangement of hepatocytes. These findings suggest that supplementation with recombinant Lactobacillus strains can effectively ameliorate the adverse effects of a high-fat diet on liver and adipose tissues (Figure 7B). 

## 4. Discussion

The long-term consumption of an HFD is known to trigger sustained and systemic low-grade inflammation, subsequently elevating the risk of developing type 2 diabetes, cardiovascular disease, gastrointestinal diseases, neurodegenerative disease, and certain types of cancer in humans [32,33]. The initial alteration in gut microbiota mediated by HFD, coupled with its direct effects on intestinal cells, constitutes a critical step towards the development of chronic systemic inflammation [34]. Amuc_1100, a pili-like protein abundantly present in *A. muciniphila*, has been identified for its capacity to induce the production of immune-related cytokines through the activation of Toll-like receptor (TLR) 2 and TLR4 signaling pathways. [15,16,17]. These studies underscore the role of Amuc_1100 in regulating immunological homeostasis at the gut mucosa and in maintaining the integrity of the gut barrier [35,36]. 

Given that Amuc_1100 is a membrane protein, its recombinant expression on the cell surface is, therefore, pivotal for the full realization of its biological functions. Strategies for displaying heterologous proteins on the surface of lactic acid bacteria encompass the use of N-terminal transmembrane anchors, lipoprotein anchors, covalent anchoring to the cell wall via LPXTG motifs, and non-covalent associations through binding domains [37]. The employment of LPXTG domain anchors has been shown to enhance the interactions between host cells and target proteins, thereby augmenting the therapeutic effects [38,39,40]. Importantly, there exists considerable species-specific variation in the LPXTG motif responsible for sortase-mediated anchoring in Lactobacilli. Consequently, in this study, the functional Amuc_1100 was expressed on the cell surface of *L. rhamnosus* and *L. plantarum* using host-derived signal peptides and LPXTG motif anchors, respectively.

Tight junction proteins are closely regulated, which play a pivotal role in maintaining gut barrier integrity [41]. Several transmembrane and cytosolic proteins, such as zonula occludens (ZOs), claudins, occludin, and junctional adhesion molecules, are used to construct tight junction complexes [42]. ZOs are crucial for cytoskeleton formation, whereas claudins regulate the paracellular space. The downregulation of these proteins exacerbates intestinal permeability, facilitating the leakage of gut microbiota-derived LPSs and other toxins into the bloodstream. This process creates a deleterious cycle, contributing to insulin resistance and obesity [43]. It is evident that the supplementation with a single probiotic strain does not fully mitigate the damage induced by an HFD. Moreover, the continuous administration of certain probiotic strains can lead to a decline in other probiotic populations, as the diet can swiftly alter the composition of the gut microbiota [18]. Therefore, the construction of an integrated, colonizable probiotic strain capable of producing regulatory metabolites and expressing various functional proteins, informed by the multiple-omics analysis of gut microbiota, the functional investigation of microbial proteins, and the usage of advanced biotechnology, is a promising strategy.

The selection of appropriate chassis hosts constitutes both a critical and challenging step in microbial engineering. Despite the extensive study and widespread application of Lactobacillus strains, significant variability in the efficacy of recombinants expressing identical proteins persists. In this investigation, the recombinant strain pLR-GAA-H demonstrated superior intestinal barrier protection in comparison to pLP-GAA-H when challenged with an HFD. This discrepancy is likely attributable to the unique metabolites and secreted proteins of the host strains, which may synergize with AMUC_1100 to exert protective effects on host cells [44,45]. Interestingly, the improvement with higher doses of recombinant strains is better than that with lower doses. Meanwhile, this effect of recombinant strains is greater than that of the wild-type hosts at the same therapeutic dose. These findings suggest that the amelioration of high-fat diet-induced obesity is contributed to synergistically by Amuc_1100 and Lactobacillus strain. Elucidating the precise molecular mechanisms underlying these observations necessitates further experiments.

The gut microbiota plays a crucial role in human health. However, the complexity of microorganism populations, especially the majority of unculturable species, has hindered the development of microbiota-based drugs [46]. Recently, more and more probiotics have been synthetically engineered in a gain-of-function manner and utilized in the treatment of obesity, diabetes, and colitis in animal models [47]. A *Laccococtus lactis* strain producing IL-10 has even reached phase I clinical trials [48]. Hence, the ideal engineered probiotics in the future should possess the capability to target multiple points in a signal pathway, or simultaneously target multiple pathways to exert an integrated therapeutic effect.

In summary, our study has successfully engineered two recombinant Lactobacillus strains exhibiting a cell-surface display of Amuc_1100. The daily oral administration of these strains to HFD-fed adult mice resulted in a reduction in body weight gain and improvements in glucose homeostasis, attributed to the stabilization of the gut barrier and the induction of anti-inflammatory cytokines. This study provides a method for developing recombinant strains capable of expressing a variety of probiotic functional proteins and a feasible approach for the clinical treatment of glucose homeostatic disorders through the use of bioengineered probiotics.

## Data Availability

The original contributions presented in the study are included in the article/Supplementary Materials, and further inquiries can be directed to the corresponding author/s.

## Supplementary Materials

The following supporting information can be downloaded at https://www.mdpi.com/article/10.3390/bioengineering11060574/s1: Supplementary Table S1: The list of materials; Supplementary Table S2: The list of RT-PCR primers; Supplementary Table S3: The sequence of Amuc_1100 fusion protein with the LPXTG motif of *L. rhamnosus*; and Supplementary Table S4: The sequence of Amuc_1100 fusion protein with the LPXTG motif of *L. plantarum*.
