# Supplementary material for: The Recombinant Lactobacillus Strains with the Surface-Displayed Expression of Amuc_1100 Ameliorate Obesity in High-Fat Diet-Fed Adult Mice
Source: Bioengineering (Basel). 2024 Jun 6;11(6):574. doi: 10.3390/bioengineering11060574 (PMC11200897; doi:10.3390/bioengineering11060574)
Supplement: Supplementary file 1 [file bioengineering-11-00574-s001.zip › bioengineering-2981263-supplementary.pdf]

Supplementary Table S1. The list of materials

Product Name	Catalog Number	Manufacturer/Company
plasmid pTRK892	71803	Addgene
Lactobacillus rhamnosus GG (ATCC 53103)	CICC 6141	China center of industrial culture collection (Beijing, China)
Lactobacillus plantarum (ATCC 8014)	GDMCC 1.140	Guangdong microbial culture collection center (Guangdong, China)
Escherichia coli DH5a	EC0111	Thermofisher Scientific
Escherichia coli MC1061	C66303	Thermofisher Scientific
Luria–Bertani broth	28320	Guangdong Huankai Microbial Sci. & Tech. Co., Ltd.
MRS broth	27312	Guangdong Huankai Microbial Sci. & Tech. Co., Ltd.
T4 DNA Ligase	M0202S	New England Biolabs.
E*co*R I	1040A	Takara Biomedical Technology Co. Ltd.
E*co*R V	1042A	Takara Biomedical Technology Co. Ltd.
Hipure Total RNA Mini Kit	R4121-03	Guangzhou Magen Biotechnology Co., Ltd.
Evo M-MLV RT Premix for qPCR	AG11706	Accurate Biotechnology(Hunan)Co.,Ltd.
Plasmid Mini Kit I	D6943	Omega Bio-Tek Inc.
RNeasy Plus Mini Kits	74134	Qiagen
RIPA lysis buffer	89900	Thermofisher Scientific Inc.
Mice standard diet	D12450B	ZhuHai Bestest Biotechnology Co., Ltd.
Mice high fat diet	D12492	ZhuHai Bestest Biotechnology Co., Ltd.
GFP antibody	66002	Proteintech Group Inc.
IL-1 Beta antibody	66737-1	Proteintech Group Inc.
IL-10 antibody	60269-1	Proteintech Group Inc.
Beta Actin antibody	66009-1	Proteintech Group Inc.
IgG (H+L), HRP	PR30012	Proteintech Group Inc.
Erythromycin	E8100	Beijing Solarbio Science & Technology Co., Ltd.
Ampicillin	A1170	Beijing Solarbio Science & Technology Co., Ltd.
Mouse Lipopolysaccharides (LPS) ELISA Kit	CSB-E13066m	CUSABIO Technology
Hematoxylin and Eosin staining kit	ab245880	Abcam

Supplementary Table S2. The list of RT-PCR primers

Genes	Forward (5'-3')	Reverse (5'-3')
Il-1β	CAACCAACAAGTGAATTCTCCATG	GATCCACACTCTCCAGCTGCA
Il-6	CACTTCACAAGTCGGAGGCT	CTGCAAGTGCATCATCGTTGT
Il-10	GGTTGCCAAGCCTTATCGGA	CCTTGATTTCTGGGCCATGC
Zo-1	AGAGACAAGATGTCCGCCAG	TGCAATTCCAAATCCAAACC
Cldn-1	GGGGACAACATCGTGACCG	AGGAGTCGAAGACTTTGCACT
Rpl-19	GAAGGTCAAAGGGAATGTGTTCA	CCTTGTCTGCCTTCAGCTTGT

Supplementary Table S3. The sequence of Amuc_1100 fusion protein with the LPXTG motif of *L. rhamnosus**

MRLFGEEKTRYRLYKSGKLWLVALIGVFALAIGHQPSHVKASSVDMSKGEELFTGVVPILVELDGDVNGHKFSVSGEGE
GDATYGKLTLKFICTTGKLPVPWPTLVTTFAYGVQCFSRYPDHMKRHDFFKSAMPEGYVQERTIFFKDDGNYKTRAEVK
FEGDTLVNRIELKGIDFKEDGNILGHKLEYNYNSHNVYIMADKQKNGIKVNFKIRHNIEDGSVQLADHYQQNTPIGDGP
VLLPDNHYLSTQSVLSKDPNEKRDHMVLLEFVTAAGITHGMDELYKGGGSGGGSMSNWITDNKPAAMVAGVGLLLFL
GLSATGYIVNSKRSELDKKISIAAKEIKSANAAEITPSRSSNEELEKELNRYAKAVGSLETAYKPFLASSALVPTTPTAFQNE
LKTFRDSLISSCKKKNILITDTSSWLGFQVYSTQAPSVQAASTLGFELKAINSLVNKLAECGLSKFIKVYRPQLPIETPANN
PEESDEADQAPWTPMPLEIAFQGDRESVLKAMNAITGMQDYLFTVNSIRIRNERMMPPPIANPAAAKPAAAQPATGAAS
LTPADEAAAPAAPAIQQVIKPYMGKEQVFVQVSLNLVHFNQPKAQEPSEDTRGGGSPTTPETSTVPSTSSQSATTEVITPS
AQRRLPNTNEKHEYGIAAVGLALLSLMGLGSTLLFRKAKRQ

* The capital letters with the background of grey, green, bright blue and pink represent the sequences of signal peptide, GFP, Amuc_1100 and LPXTG motif, respectively.

Supplementary Table S4. The sequence of Amuc_1100 fusion protein with the LPXTG motif of *L. plantarum*[#]

MRRKLVGYMLSMLTVILALFMLGSTAHAKEVDMSKGEELFTGVVPILVELDGDVNGHKFSVSGEGEGDATYGKLTLKF
ICTTGKLPVPWPTLVTTFAYGVQCFSRYPDHMKRHDFFKSAMPEGYVQERTIFFKDDGNYKTRAEVKFEGDTLVNRIEL
KGIDFKEDGNILGHKLEYNYNSHNVYIMADKQKNGIKVNFKIRHNIEDGSVQLADHYQQNTPIGDGPVLLPDNHYLSTQ
SVLSKDPNEKRDHMVLLEFVTAAGITHGMDELYKGGGSGGGSMSNWITDNKPAAMVAGVGLLLFLGLSATGYIVNSKR
SELDKKISIAAKEIKSANAAEITPSRSSNEELEKELNRYAKAVGSLETAYKPFLASSALVPTTPTAFQNELKTFRDSLISSCK
KKNILITDTSSWLGFQVYSTQAPSVQAASTLGFELKAINSLVNKLAECGLSKFIKVYRPQLPIETPANNPEESDEADQAPW
TPMPLEIAFQGDRESVLKAMNAITGMQDYLFTVNSIRIRNERMMPPPIANPAAAKPAAAQPATGAASLTPADEAAAPAAP
AIQQVIKPYMGKEQVFVQVSLNLVHFNQPKAQEPSEDTRGGGSPVTEPGTTEPSKPGEPGTTEPSKPGEPGTTEPSKPGEP
GTTEPSKPGEPGTTEPSKPGEPGTTEPSQPGEPGTTEPSKPDEPGTTEPSQPGKPGKPGEPGTTEPGNPGTTGPTAPQPERP
AVPGPSQPAAPKPGQSGLGQPALPGLIKQPSTGVNGAGGTVGNGVTTGMNGFGTPTGSDQSTSAGYNHGTLPQTSEKQS
PIWVIFAGLLGLLIAAVGIGYRRRA

[#] The capital letters with the background of grey, green, bright blue and pink represent the sequences of signal peptide, GFP, Amuc_1100 and LPXTG motif, respectively.
